# B-factor prediction in proteins using a sequence-based deep learning model

**DOI:** 10.1016/j.patter.2023.100805

**Published:** 2023-08-04

**Authors:** Akash Pandey, Elaine Liu, Jacob Graham, Wei Chen, Sinan Keten

**Affiliations:** 1 Department of Mechanical Engineering, Northwestern University, Evanston, IL, USA; 2 Department of Civil and Environmental Engineering, Northwestern University, Evanston, IL, USA

## Abstract

B factors provide critical insight into protein dynamics. Predicting B factors of an atom in new proteins remains challenging as it is impacted by their neighbors in Euclidean space. Previous learning methods developed have resulted in low Pearson correlation coefficients beyond the training set due to their limited ability to capture the effect of neighboring atoms. With the advances in deep learning methods, we develop a sequence-based model that is tested on 2,442 proteins and outperforms the state-of-the-art models by 30%. We find that the model learns that the B factor of a site is prominently affected by atoms within a 12–15 Å radius, which is in excellent agreement with cutoffs from protein network models. The ablation study revealed that the B factor can largely be predicted from the primary sequence alone. Based on the abovementioned points, our model lays a foundation for predicting other properties that are correlated with the B factor.

## Highlights

• A sequence-based learning model is developed for the prediction of the B factor proteins
• It is shown that the primary sequence is sufficient for the B factor prediction
• Cutoff radius of 15 Å is obtained based on the developed model

## The bigger picture

B factor is a measure of atom’s displacement about their mean position, and it provides critical insights into the flexibility, solvent accessibility, thermal stability, and activity of the region in protein. In this work, we develop a sequence-based deep learning model that can predict the B factor of C_α_ atoms in proteins. The developed model is tested on 2,442 proteins, and it outperforms the state-of-the-art model by 30%. Using the developed model, we find that the B factor of a site is prominently affected by atoms within a 12–15 Å radius and in excellent agreement with cutoffs based on protein network models. In the future, this model can be used for the prediction of mechanical properties of protein-based materials, identifying active regions in the protein for chemical as well as pharmaceutical applications. Moreover, since our model can predict B factor just based on the primary sequence, it can be used to assist the design of *de novo* proteins for specific applications.

## Teaser

B factor of C_α_ atoms in proteins is an important indicator of various properties in proteins. Hence, in this work, we develop a sequence-based deep learning model to predict the B factor and test its robustness. We then use the developed model to extract some physically relevant information about the B factor and the factors that influence it.

## Introduction

The B factor, also known as the Debye-Waller factor or temperature factor, is an important property of the atoms in protein signifying the displacement of atoms about their mean position. Experimentally, it is defined as the attenuation of X-ray scattering; the lower the attenuation rate, the higher the B factor. In the literature, the B factor has been used as the indicator of protein’s flexibility and dynamic properties.^1^^,^^2^ In addition, the B factor has also been used to develop structural bioinformatics,^3^ identify the active regions,^4^ and study the thermal stability^5^ of proteins. More broadly, B factor and Debye-Waller factor are profoundly important parameters for understanding soft matter physics related to glass formation, dynamical heterogeneity, and mechanical behavior, as well as training and validating multiscale models.^6^^,^^7^^,^^8^ In this sense, for understanding protein dynamics and how it relates to functions, it is critically important to learn both what governs the B-factor values and how to predict them in the absence of experimental data.

There has been lots of progress over the past few decades in physics-based models for reproducing the B factors of proteins and these methods need structural information of proteins. A common idealization is to model proteins as bead-spring systems with elastic spring constants tailored to match fluctuations or distance-based criteria. This is based on the premise that, for each atom, the B factor is highly dependent on its interaction with the neighboring atoms, with nearby atoms having a greater role as they tend to have stronger physical interactions with the atom. Normal mode analysis (NMA) uses a Hamiltonian matrix for atomic interactions and eigenvalues of the system are correlated with the B factors.^9^^,^^10^^,^^11^^,^^12^ The anisotropic network model was proposed to simplify NMA by using a one-parameter spring interaction potential and was still able of capturing the important features of NMA.^6^^,^^13^ In a similar vein, a Gaussian network model^14^ uses the Kirchhoff matrix to depict the interaction between alpha carbon ($C_{\alpha }$) atoms, offering advantages in computational efficiency relative to NMA. More recently, the use of flexibility and rigidity (FRI) methods,^15^ where the interaction graphs are generated based on radial basis functions, improved the prediction of the B factor. Various versions of FRI were introduced to make the method faster and more feasible to predict anisotropic motion and capture multiscale interactions.^16^^,^^17^^,^^18^

Physics-based models are advantageous as they offer important insights for a specific protein. However, they need structural information of the protein and do not generalize well outside the training dataset.^19^ Several approaches have tried to address the shortcomings of physical models with machine learning (ML) techniques.^20^^,^^21^^,^^22^ One of the state-of-the-art (SOTA) models for predicting B factors is based on multiscale weighted colored graphs (MWCGs).^23^ The MWCG method generates three 2D matrices (channels) for each atom in the protein based on its interaction with the heavy atoms carbon, nitrogen, and oxygen. These three channels of data are then combined with global features based on the quality of the atomic model obtained from crystallographic data in the Protein Data Bank (PDB), namely the R value, and resolution for the prediction of the B factor. These transformed features are then fed to a convolution neural network for prediction. The authors^19^ tested the MWCG on 364 proteins using a leave-one-out strategy and obtained an average Pearson correlation coefficient (PCC) of 0.66, considering only $C_{\alpha }$ atoms for predictions.

In previous studies, it has been reported that the B factor is not an absolute property as its magnitude depends on factors such as degree of resolution, solvent content, and overall quality of data,^24^ which can lead to errors or differences in reported B-factor values for a given protein. A more reliable approach to getting insight into the dynamics of different regions is to normalize the B-factor data for each protein before comparing any two proteins. Normalized B factor is regularly used in various computational analyses as well as protein crystallography^2^ and is a better choice for calibrating and validating ML models. A couple of ML approaches that focused on predicting normalized B factors with methods such as support vector regression (SVR) have reported PCC in the range of 0.53–0.61. The test datasets^25^^,^^26^ reported were limited in size, encompassing roughly 300–800 proteins. Given that today we have approximately 192k proteins in the PDB, it is important to test any proposed model on a large test dataset to demonstrate generalizability. In addition, in these methods, input features of all the atoms are mostly feature-engineered, i.e., the embedding for each residue in the protein is generated by searching for the multiple sequence alignment using PSI-BLAST.^27^ It would be more desirable to have a model that can access the whole protein simultaneously and predict the B factors of all the $C_{\alpha }$ atoms with minimum feature engineering. Last, but not the least, with the advancements in the area of deep learning (DL) models, we envision that the PCC can be improved further. To help address these issues, here we present a DL model employing a long short-term memory (LSTM) network to predict the B factor as well as the normalized B-factor values of any protein. The key elements of our contribution include:

(1) The use of a sequence-based DL model (LSTM) for the prediction of the B factor.
(2) The broadest testing dataset comprised of 2,442 proteins for demonstrating generalizability.
(3) Systematic studies to identify a minimalist approach to predicting protein fluctuations with greater efficiency, accuracy, and without empirical input. This will involve the analysis of the relative importance of primary sequence (PS), secondary structure (SS), $C_{\alpha }$ atom coordinates (CoI), and chain information (ChI) for the prediction of the B factor.
(4) Systematic studies to quantify the radius within which one atom influences the B factor of another atom significantly (e.g., a cutoff threshold).

## Results and discussions

### Prediction of normalized B factor

**Figure 1:**
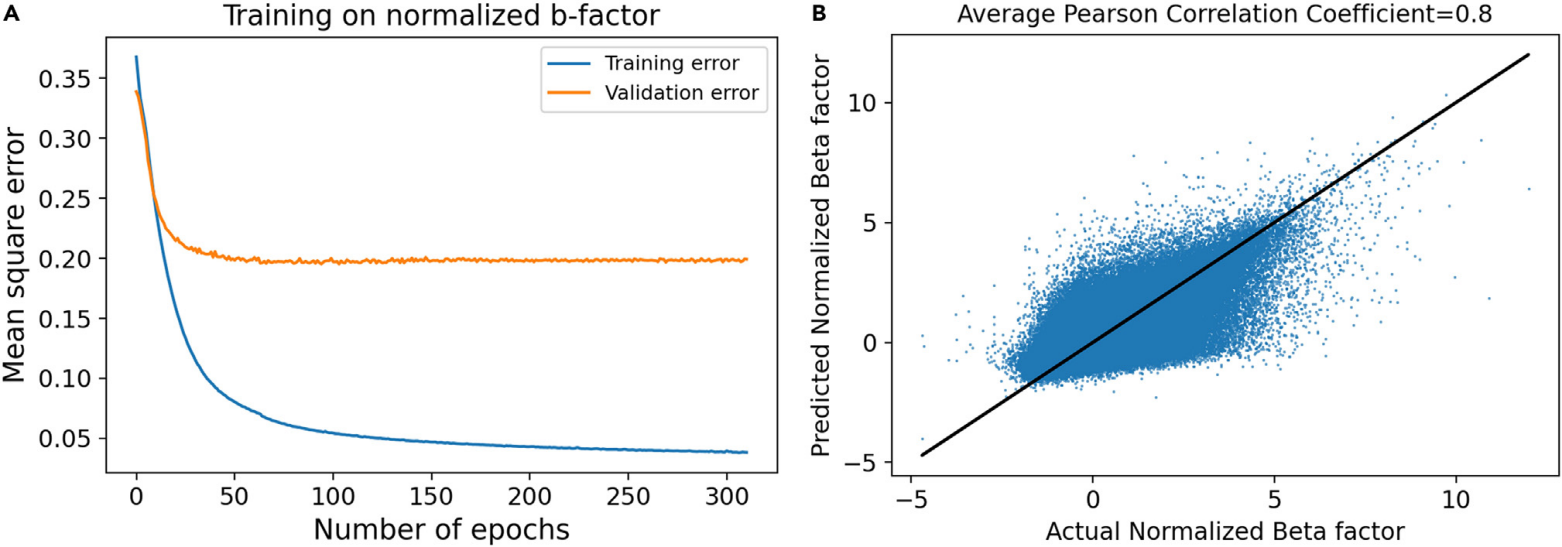
Deep learning model training and testing details (A) Training and validation error trend for normalized B factor model. (B) Predicted versus actual normalized B factor of atoms in 2.4k proteins in the test dataset.

In this section, we present the results for the prediction of normalized B-factor values using the method described in experimental procedures. Before training and testing, the B-factors of all proteins are normalized using Equation 5a. The training and validation error is shown in Figure 1A and it shows that the change in the validation error after 200 epochs is negligible. This also demonstrates that the model does not overfit the data, which would manifest in an increase in the validation error with the increasing number of epochs. The model optimized is tested on 2.4k proteins and the comparison between the predicted normalized B factor and actual normalized B-factor graph is shown in Figure 1B. The averaged PCC, which is used for checking the quality of the fit, is calculated to be 0.8 on the test dataset. As pointed out in experimental procedures, to check the robustness of the model, it was trained and tested using four different seeds, and we observed minimal variation in the average PCC between 0.795 and 0.80. To the best of our knowledge, the PCC of 0.8 for the normalized B factor is the highest ever reported in the literature. At the time of writing this paper, the average PCC values reported by the SOTA models^25^^,^^26^ are between 0.53 and 0.61. Both of the SOTA models use an SVR technique and report the average PCC within a similar range. For the training/prediction of the normalized B factor of an atom in a protein, SOTA models used the PS information in window sizes of 9 and 15. For one-on-one comparison, we trained our model without the proteins that were used for testing the SOTA model^25^ as the data were easily available. We obtain an average PCC of 0.7 which is 30% higher than the SOTA average PCC of 0.54.

### Importance of input features

**Figure 2:**
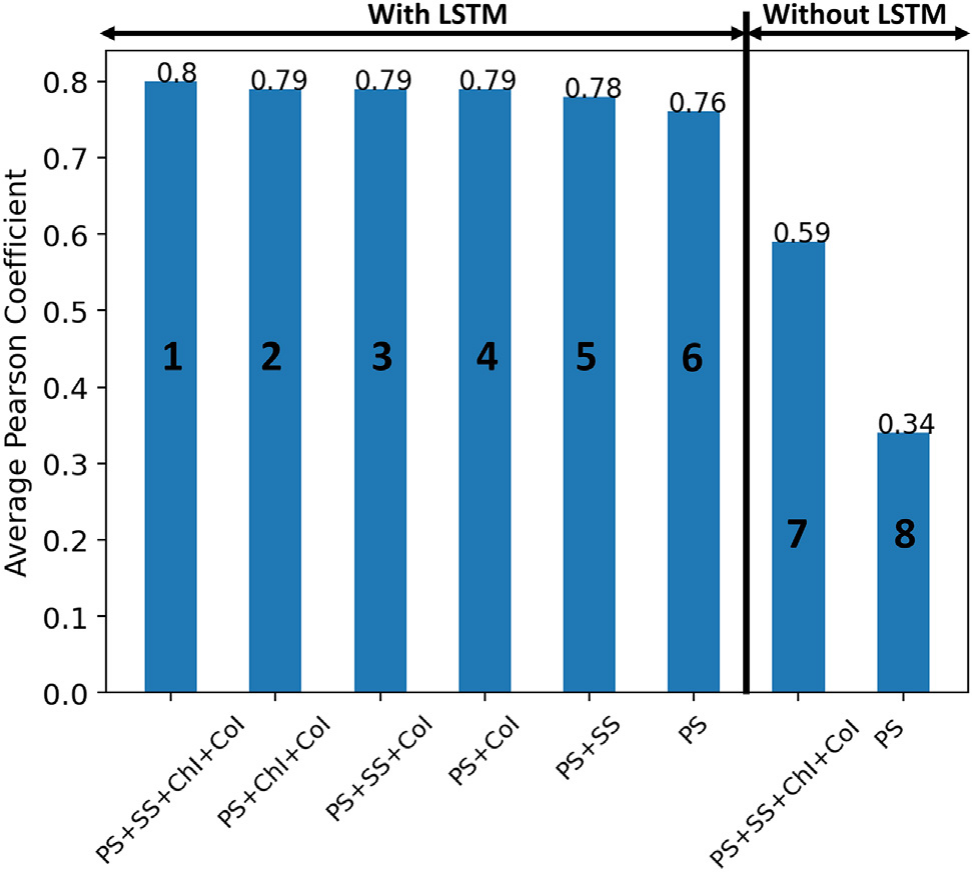
Impact of various input features on average PCC The above acronyms are expanded as follows: PS, primary sequence; SS, secondary structure information; ChI, chain information; CoI, $C_{\alpha }$ coordinate information; and LSTM, long short-term memory.

The results presented until now use PS, SS information, CoI, and the start/end of ChI as input features. But, as in the case of any ML model, some features are more important than others. In this section, we examine the importance of each feature. To study the importance of a feature, it is just removed from the input features and the whole model is trained and tested again. Models with different features are considered for this study and their details and results (average PCC) are shown in Figure 2.

The x-label in Figure 2 indicates which input features are included in the model. Comparing models 2, 3, and 4, it is evident that models that include PS and CoI exhibit indistinguishable PCC scores. The comparison of model 6 with models 4 and 5 indicates that the addition of CoI and SS to PS results in the increase of PCC score by 3.5% and 2.5%, respectively. This comparison places CoI above SS in the importance matrix. At the same time, when only PS is considered, as in model 6, the <5% drop in the PCC score compared with models 1–5 is marginal. For many proteins, PS is the only known feature, so developing a strong predictive algorithm that depends minimally on CoI, SS, and ChI is a critical contribution of this work. This also points to the fact that, if the DL model is robust, it can learn structural information such as coordinate information^20^ and SS^28^ based only on the PS. In addition, the developed model can offer insights into designing *de novo* proteins using *in silico* models, which mostly take only the PSs as the input.

**Figure 3:**
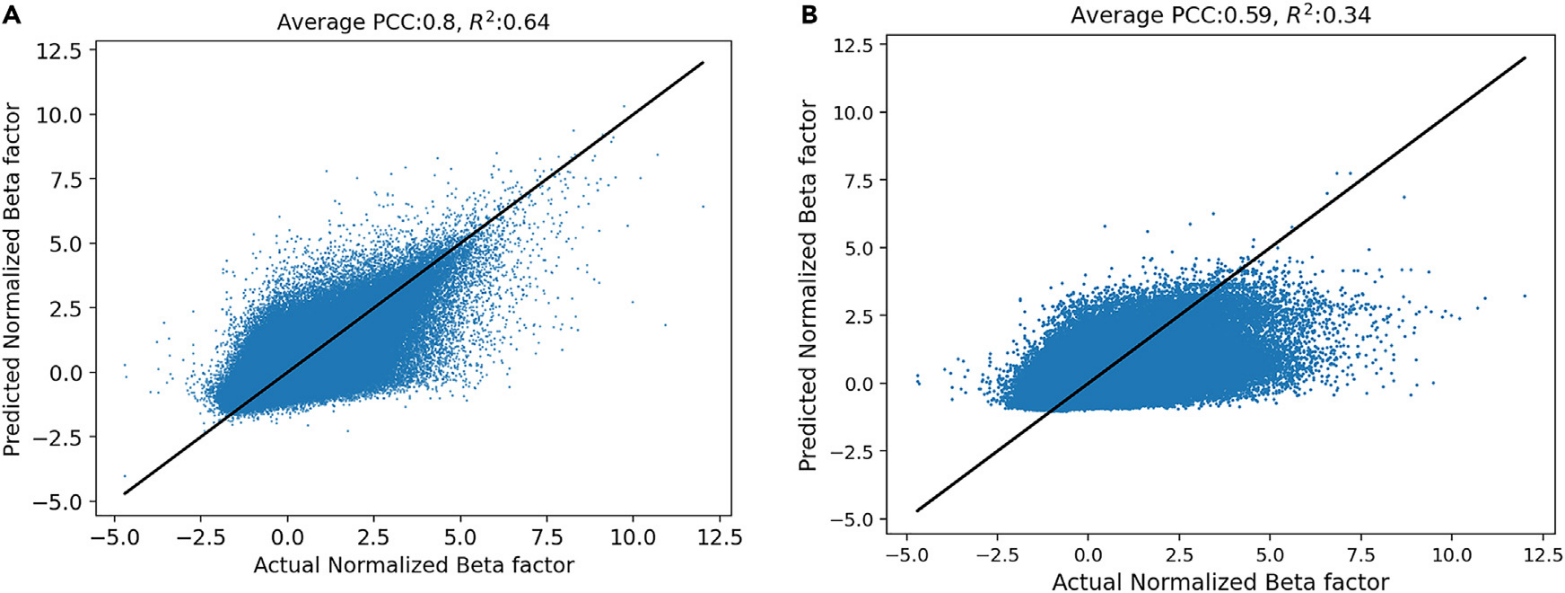
Quality of fit with and without LSTM (A) Actual versus predicted normalized B factor on the test dataset when trained with all input features with LSTM (model 1). (B) Actual versus predicted normalized B factor on the test dataset when trained with all input features without LSTM (model 7).

Models 7 and 8 were run to check the importance of LSTM in our model. In model 7, all possible input features are used without the LSTM and, in model 8, only the PS feature is used without LSTM. Removing the LSTM from the model transforms it into a simple feedforward neural network model. The only difference in models 6 and 8 is the absence of LSTM, which leads to a drastic drop of 55% in the average PCC. This emphasizes that PS information without LSTM is inadequate for the prediction of the B factor. This also informs us that the B factor depends not only on amino acid type but also on $d_{seq}$ and $d_{euc}$. The extent of this impact is discussed in result interpretability. Models 1 and 7 use all input features, but model 7 lacks LSTM. The PCC score in model 7 is 0.59, which is 26% lower than model 1, but 73% higher than model 8. The difference in the PCC score of models 7 and 8 is attributed to the fact that SS and CoI in model 7 inform the DL model about the atom’s surroundings. To check if in model 7 the model has learned something meaningful, we look at the actual versus predicted $\hat{B_{i}}$ in Figure 3. The difference in the $R^{2}$ value of models 1 and 7 shows that model 7 model lacks generalizability. Based on these observations, it can be stated that neighboring atoms prominently influence the B factor and that the LSTM-based model is sufficient to capture those impacts.

We also want to stress that, out of all the models that use LSTM (models 1–6), only model 6 is a purely PS-based model as it does not use any structural information about the protein. Models 1–5 are hybrid sequence- and structural-based models as they use some form of structural information for the prediction.

### Result interpretability

Based on the validation of the model on our test dataset and its superior performance relative to SOTA models, we conclude that our model is robust. In this section, we use this model to extract some meaningful information such as the impact of $d_{seq}$, $d_{euc}$, type of amino acid, and type of SS on the prediction of ${\hat{B}}_{i}$.

#### Impact of window size $W_{s}$

**Figure 4:**
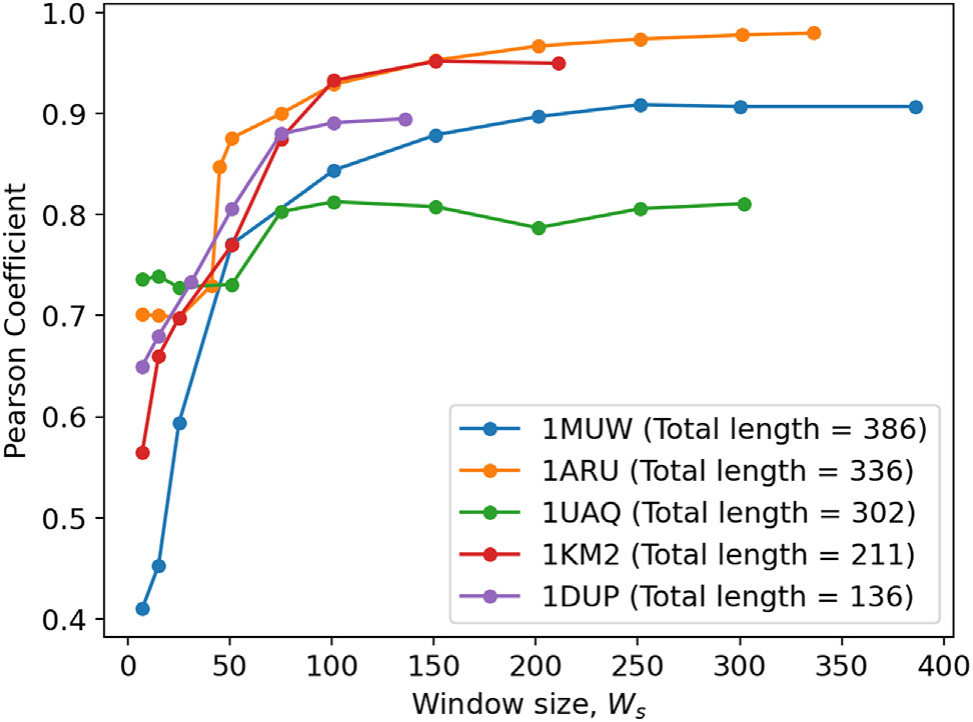
Effect of window length $W_{s}$ on B-factor prediction Effect of variation of PCC, of five proteins of varying sequence length, as a function of $W_{s}$ considered for the prediction.

As pointed out in experimental procedures, to study the effect of $d_{seq}$ in the PS, we first study the impact of window size $W_{s}$ on the PCC. To do so, we randomly selected five proteins (1MUW, 1ARU, 1UAQ, 1KM2, and 1DUP) and used our trained model to predict $\hat{B_{i}}$ by considering different $W_{s}$ values. The trend of the PCC with respect to the $W_{s}$ considered for prediction is shown in Figure 4 for all five proteins. It can be seen that, for all five proteins analyzed, PCC increases sharply with $W_{s}$ up to the critical value of $W_{s}^{c}$ and, for $W_{s}$ > $W_{s}^{c}$, the PCC remains nearly invariant. The data indicate that the accuracy of the model converges above $W_{s}^{c}$ and that $W_{s}^{c}$ is specific to each protein, ranging from 45 for 1ARU to 70 for 1KM2. Previously the models have used $W_{s}$ up to 15 for $\hat{B_{i}}$ prediction.^25^^,^^26^ We suspect that this value might be too low and this would explain the lower PCC values attained with the SOTA methods. Since $W_{s}^{c}$ is not the same for all the proteins, it is difficult to make one fully connected neural network that will perform best for all the proteins. This justifies the use of a sequence-based model (LSTM) in this study as it can deal with the varying *N* without changing the number of parameters in the model. We also note from Figure 4 that for some proteins PCC versus $W_{s}$ is sigmoidal as the PCC value plateaus at low $W_{s}$ but, for others, this trend is not observed.

#### Estimating cutoff radius $R_{cut}$

Figure 4 demonstrates the limits of $d_{seq}$ on predictive accuracy since PCC does not change significantly beyond $W_{s}^{c}$. However, to correctly predict $\hat{B_{i}}$, it is also important to get a clear idea about the impact of $d_{euc}$. Before presenting the results, we declare a variable called cutoff radius $R_{cut}$, which indicates that for the calculation of $\hat{B_{i}}$ we only consider the impact of atoms that are at $d_{euc}$ ≤ $R_{cut}$.

**Figure 5:**
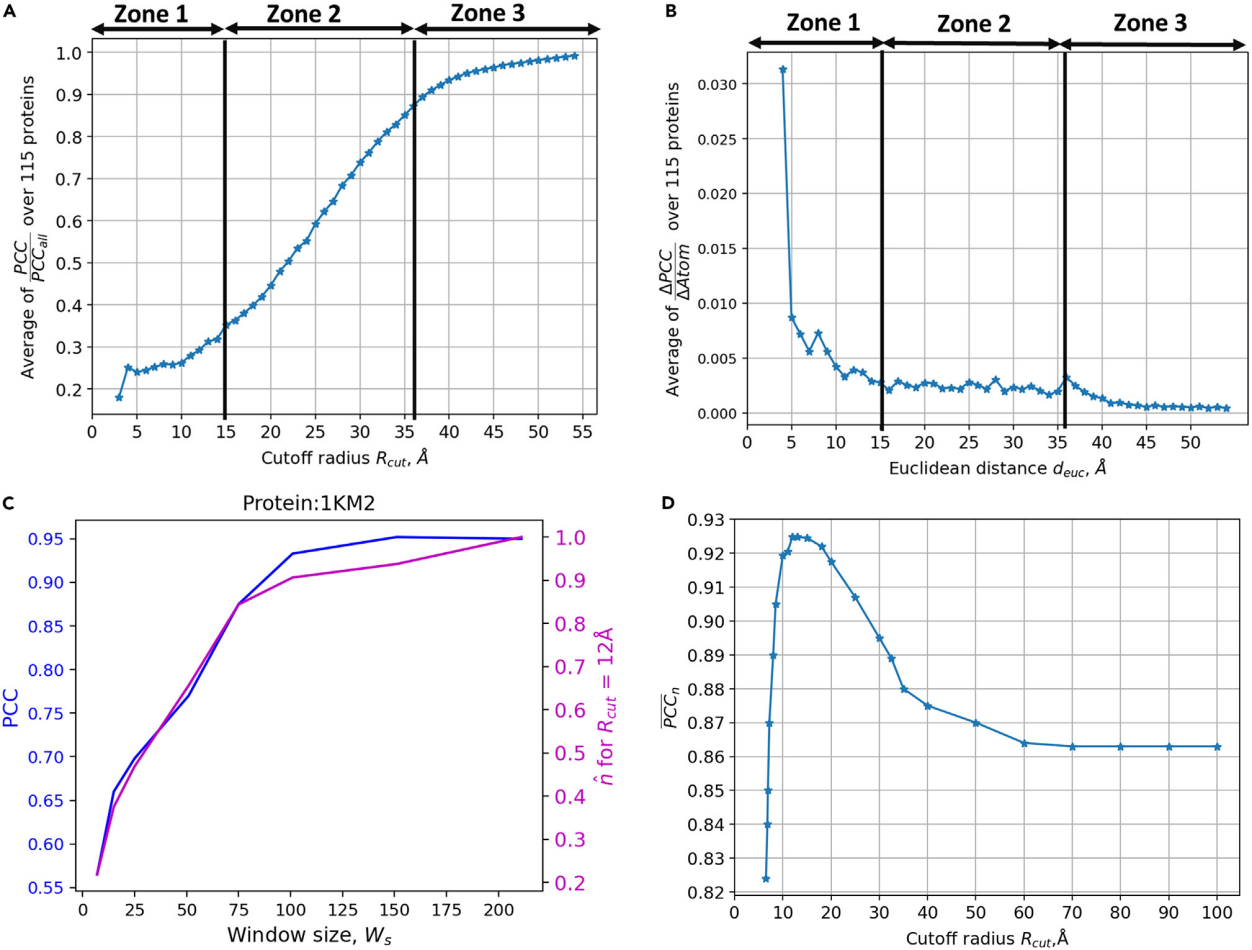
Identifying a cutoff radius $R_{cut}$ (A) $\frac{PCC}{PCC_{all}}$ with respect to $R_{cut}$. (B) Variation of $\frac{\Delta PCC}{\Delta Atoms}$ with respect to the cutoff $d_{euc}$. (C) Variation of PCC and $\hat{n}$ ($R_{cut}$ = 12 Å) with respect to $W_{s}$. (D) Variation of ${\bar{\text{PCC}}}_{n}$, comparing the trend of the blue and orange line in (C), with respect to $R_{cut}$.

As discussed in algorithm to study the impact of neighboring atoms, we first plot the variation of $\frac{PCC}{PCC_{all}}$ with respect to $R_{cut}$ in Figure 5A, where $PCC_{all}$ is the PCC of the protein when all the atoms in the protein are considered for the calculation of $\hat{B_{i}}$. From Figure 5A, we observe that $\frac{PCC}{PCC_{all}}$ increases with the increase in $R_{cut}$. As the number of atoms considered for the calculation of the B factor increases, the PCC of the protein also increases and converges to the value of $PCC_{all}$. However, Figure 5A does not give a clear idea about the relative importance of vicinal atoms and whether there is a cutoff distance that can be identified. Therefore, as discussed in the algorithm section, we plot the variation of $\frac{\Delta PCC}{\Delta Atoms}$ with respect to the Euclidean distance $d_{euc}$ to find the contribution per atom at various $d_{euc}$ in Figure 5B. Based on the variation of $\frac{\Delta PCC}{\Delta Atoms}$ in Figure 5B, we identify three zones of influence for $\hat{B_{i}}$. Zone 1 is in between 0 and 15 Å and atoms in this zone contribute the most in the prediction of $\hat{B_{i}}$. Zone 2 is in between 15 and 36 Å and the atoms in this zone contribute approximately equally to the prediction of $\hat{B_{i}}$. Anything beyond 36 Å is defined as zone 3 and the contribution from this zone is nearly negligible.

Based on the above observations, we conclude that atoms generally lying within ≈15 Å of each other are more likely to impact one another more profoundly than atoms further out. To further strengthen the claim of this result, we carry out a second study regarding $R_{cut}$. To clarify this analysis we first introduce a variable called the normalized number of $C_{\alpha }$ Atoms ($\hat{n}$) that lie within a given cutoff distance $R_{cut}$ from an atom. To calculate $\hat{n}$ for a $R_{cut}$ value, for each of the *N* amino acids in a protein, first, the average number ($\bar{n}$) of $C_{\alpha }$ atoms within the cutoff radius is calculated using the Equation 1a, where $n_{i}$ is the number of $C_{\alpha }$ atoms within the cutoff radius $R_{cut}$ of the $i^{th}$ $C_{\alpha }$ atom. Then, $\hat{n}$ is calculated using Equation 1b.

(Equation 1a) $$\bar{n}=\frac{\sum_{i=1}^{N}n_{i}}{N}$$

(Equation 1b) $$\hat{n}=\frac{\bar{n}}{N-1}$$

As part of the second study, we first calculate $\hat{n}$ as a function of $W_{s}$ for 23 different proteins from the test dataset with *N* varying between 60 and 500. The variation of $\hat{n}$ and PCC with respect to $W_{s}$ for protein 1KM2 is shown in Figure 5C, from which we observe that there is some correlation between $\hat{n}$ and the PCC curve. To quantify this correlation, we calculate the Pearson correlation coefficient between the $\hat{n}$ curve (magenta curve) and the PCC curve (blue curve) in Figure 5C. Let us indicate this Pearson correlation coefficient as PCC_*n*_. Since, $\hat{n}$ is the function of the cutoff distance $R_{cut}$, the PCC_*n*_ is also the function of $R_{cut}$. Therefore, we calculate PCC_*n*_ for different $R_{cut}$ values for all 23 proteins. The trend of averaged PCC_*n*_ (${\bar{\text{PCC}}}_{n}$) with respect to $R_{cut}$ is shown in Figure 5D. It can be observed that ${\bar{\text{PCC}}}_{n}$ first increases with $R_{cut}$ and then decreases after reaching a maximum value around the $R_{cut}$ of 12–15 Å. Based on the study in Figure 5, we claim that the atoms within 15 Å of Euclidean distance have the greatest impact per atom on B-factor calculation, but information of atoms at further distances is still necessary to achieve a high PCC.

#### Impact of the type of amino acid on the normalized B factor

**Figure 6:**
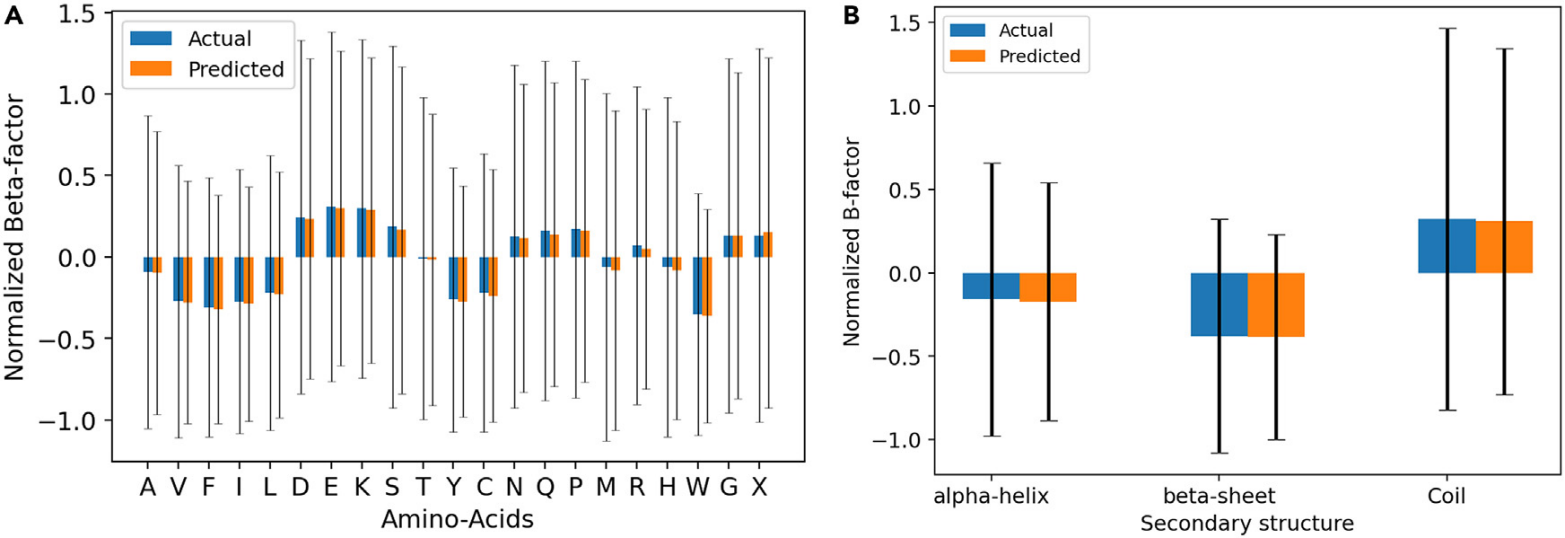
Impact of type of the amino acid and the secondary structure on the B factor (A) Variation of normalized B factor with respect to the amino acids. (B) Variation of normalized B factor with respect to the secondary structures.

From the above studies, we observed that the B factor of an atom is highly dependent on $W_{s}$ and $d_{euc}$. To provide more insight into how chemical details might influence B factors, we turn our attention to the influence of amino acid type on B-factor prediction. For this purpose, we plotted the mean normalized B factor (actual and predicted), with an error bar, for every amino acid using the test dataset. The plot is shown in Figure 6A and it can be observed that the mean predicted normalized B-factor variation with respect to the amino acid follows the same trend as the mean actual normalized B factor. However, it can also be seen that the standard deviation is consistently under-predicted as our developed model predominantly under-predicts the B factor, which is evident from Figure 1B. B-factor standard deviations are shown in Figure 6A to quantify the breadth of the distribution of both predicted and actual values when clustered for each type of amino acid. Given that the amino acid type alone does not accurately describe its B factor, we observe large standard deviations in both the actual and the predicted results. This is because the B factor of an amino acid is highly dependent on its position in the 3D protein structure. This fact is further emphasized by our study in Figure 5 that the atoms within 15 Å radius have the greatest impact on each other’s B factor. Therefore, the primary contributor to the B factor of a residue in a protein is its location and vicinal residues. The chemical nature of the residue does influence its own B factor, but it is not the sole contributor. For the same reason, when we predict the B factor of an amino acid based on its type and without an LSTM in model 8 in Figure 2, this results in the lowest PCC value.

Even though the standard deviations are high, we can make a couple of observations about the impact of amino acid type on the B factor.

• Hydrophobic types of amino acids such as A, V, F, I, and L mainly exhibit lower B factor as they are mostly buried within the densely packed core of the protein. Due to the densely packed core, the region is quite ordered; hence the smaller B factor. This is corroborated by the fact that hydrophobic amino acids pack into the core to form β sheet and α helix SSs predominantly. These SSs are more ordered and typically exhibit lower B factor as shown in Figure 6B. Similarly a study in the literature^29^ shows that hydrophobic amino acids A, V, F, I, and L do exhibit higher mean B-factor values. This trend is further supported by another study^30^ that shows that the low B-factor regions are rich in amino acids A, V, F, I, and L.
• Charged amino acids such as D, E, and K mainly exhibit higher B factor in the protein as they are mostly on the surface and predominantly form coils and sometimes α helices. Unlike β sheets and α helices, coils are disordered, thus exhibiting higher B factor as shown in Figure 6B. This trend is further corroborated by other studies, which show that charged amino acids do exhibit higher mean B factor^29^ and are more likely to be found in high B-factor regions.^30^

### Conclusion

The B factor is an important indicator of a protein’s dynamic behavior, but making a generalizable predictive model for it has been challenging. The challenge for the predictive models arises because the B factor of an atom is highly dependent on its surrounding and it is essential to capture its impact efficiently. In this work, we overcome this challenge by developing an LSTM-based DL model, a bidirectional sequence model that is capable of capturing long-range dependencies more effectively. LSTM helped in capturing the impact of $d_{seq}$ and $d_{euc}$ effectively in proteins of varying sizes without any feature engineering. The developed model when tested on 2.4k unseen proteins, resulted in an average PCC of 0.8 and 0.73 for normalized B factor and un-normalized B factor respectively.

Our analysis of the impact of each input feature on the prediction of the B factor indicates that using just the PS as the input feature is sufficient for the prediction of the B factor. This suggests that if the model is adequately robust, it can implicitly account for key structural contributors such as SS and atomic positions from the PS and B-factor data used in training. In addition, we used our tested model to study the impact of window size $W_{s}$ on the prediction and concluded that different $W_{s}$ values are suitable for different proteins. To expand our knowledge about the Euclidean distance within which atoms impact each other’s B factor, we used our model and found that within the $R_{cut}$ value of 12–15 Å the impact is prominent. Thus, our model can be used to not only effectively predict the B factor but also to extract meaningful physical information about the proteins.

In the future, this model can be used for the prediction of other mechanical properties of protein-based materials, identifying active regions in the protein for chemical as well as pharmaceutical applications. Moreover, since our model can predict B factor just based on the PS, it can be used to assist the design of *de novo* proteins for specific applications.

## Experimental procedures

The central objective of this work is to precisely predict B-factor values for $C_{\alpha }$ atoms in proteins by training a DL model on existing experimental B-factor data. Once trained, the model should be able to predict B factors of each $C_{\alpha }$ atom from provided input features of a protein that was not included in the training/validation datasets. In the scope of this work, these input features can be any particular one or any combination of PS, SS, CoI, and ChI. In this work by ChI feature, we indicate the start and end of the polypeptide chain. Key questions to address are which of these features are redundant? And what is the minimum amount of required features necessary to have reasonable accuracy in the predictions?

### Dataset

Protein data are extracted from PDB using PyPDB API,^31^ which is made to effortlessly perform an advanced search of PDB. Using PyPDB, PS, CoI, and ChI are extracted. Biotite,^32^ a python package, is used for extracting SS information. Currently, PDB has these data for 192k proteins but, in this study, we excluded some proteins based on the following criteria:

• If any $C_{\alpha }$ atom in the protein has a B factor above 80, given that such extreme values of B factor are indicative of an experimental error in data or large uncertainty.^33^
• If any amino acid in the protein has a B factor less than or equal to 0, since this is unphysical.
• If all the amino acids in the protein have the exact same B factor, as this is also unphysical.
• If the total number of amino acids in the protein exceeds 500. The number 500 was heuristically chosen to keep the cost of training and testing relatively low. After rejecting the data from PDB based on the above three criteria, approximately 70% of the remaining proteins have a total number of amino acids (*N*) below 500; hence covering more than the majority of proteins in this study.

We note that the last criterion can be revisited to see whether the model architecture proposed in this work can be used for training proteins with any number of amino acids. Since our focus is primarily on assessing the performance of sequence-based models for accurate prediction of B factors of $C_{\alpha }$ atoms, we leave the expansion of the scope to larger proteins to future work.

Based on the above conditions, our dataset contains ≈61,000 proteins. Out of these 61k proteins, 56k proteins are used for training, 2.4k are used as a validation dataset, and 2.4k are used as a test dataset. To the best of our knowledge, this is the widest dataset on which a model for the prediction of the B factor has been tested, which is important for assessing the generalizability of the model.

### DL model

Our DL algorithm for predicting the B factor of all the $C_{\alpha }$ atoms in proteins must address the question of how other residues in the protein sequence influence the B factor of a given atom. A key challenge here is that atoms that might be distant in sequence dimension may in fact be vicinal spatially, as determined by the folded structure of the protein. It is anticipated that, since B factors are most strongly affected by caging effects induced by nearby atoms, capturing these structural aspects and their relation to input sequences is indispensable for the success of the model. For this purpose, we have developed a sequence-based DL model. Sequence modeling is the modeling technique in ML that is used to analyze ordered input such as time series data. It is shown in the literature that sequence models such as gated recurrent unit and LSTM have captured long-range dependencies efficiently.^34^ Sequence length is defined as the total number of residues in a protein. Between any pair of residues, we identify two meaningful distances. The sequential distance $d_{seq}$ is defined as the distance between two residues in the PS, while the Euclidean distance $d_{euc}$ is defined as the distance between them in 3D space. It is important to capture the long-range dependencies because two residues that are distant in the PS (high $d_{seq}$) can be closer in the 3D space (low $d_{euc}$) due to the folded structure. Therefore, in the current model, we use LSTM for capturing the impact of $d_{seq}$ and $d_{euc}$ on the prediction of the B factor for each $C_{\alpha }$ atom in proteins. In addition, the protein is not a causal system, i.e., the property of any amino acid can be impacted by any amino acid in the protein depending on its position in the 3D structure. If we use unidirectional LSTM for a protein with a total of *N* amino acids, it generates the embedding for the $i^{th}$ amino acid, which is only dependent on the $1^{st}$ to $i^{th}$ amino acids. However, the embedding from the bidirectional LSTM is the concatenation of the effect from ${the\;1}^{st}$ to $i^{th}$ as well as the $N^{th}$ to $i^{th}$ amino acids due to the forward and reverse flow of information simultaneously. Hence, bidirectional LSTM makes a strong and novel case for this application.

**Figure 7:**
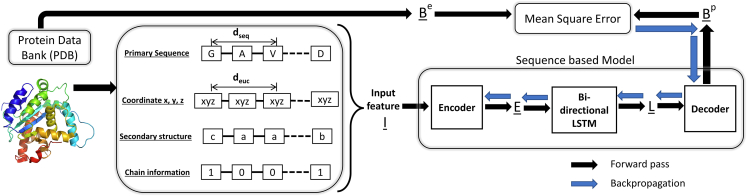
Deep learning model architecture This figure captures the process of data preparation, details of the deep-learning model, and the process of backpropagation for optimization.

The architecture of the developed DL model is shown in Figure 7. The total number of tunable parameters in the DL model for the prediction of un-normalized and normalized B factor is 3.33 and 4.65 million, respectively, as shown in Table S1 in supplemental experimental procedures. This network was finalized after fine-tuning the number of parameters in the encoder and LSTM’s hidden layer as they are critical in any sequence-based models.^35^^,^^36^ The input to the model is a sequence of input vectors where each vector $I_{i}$, of size 28 × 1, defines the $C_{\alpha }$ atom. The first 21 elements of the vector $I_{i}$ represent a one-hot encoding for a type of amino acid; 20 positions for commonly occurring amino acids and 1 for uncommon amino acids. The next 3 positions in the input vector is a one-hot encoding for the SS (β sheet, α helix, and coil structure) and the following 3 positions are for x, y, z coordinates of the $C_{\alpha }$ atom. The last element ($28^{th}$) defines the start/end of a polypeptide chain; it is 1 at a position where a chain starts/ends in the protein. Since within the elements of the vector $I_{i}$ only coordinates take on any real number other than 0 or 1, it is advantageous to normalize the coordinates. Moreover, it was noted during the development phase of the model that normalizing the coordinates, within the same protein, improved the convergence of the model. Thus, in our approach, Cartesian coordinates ($X_{i}$, $Y_{i}$, and $Z_{i}$) of each atom in a protein are normalized using the mean ($\mu_{X}$, $\mu_{Y}$, and $\mu_{Z}$) and standard deviation ($\sigma_{X}$, $\sigma_{Y}$, and $\sigma_{Z}$) of the coordinates in the same protein according to Equation 2. In Equation 2, $\hat{X_{i}}$, $\hat{Y_{i}}$, and $\hat{Z_{i}}$ are the normalized coordinates of $i^{th}$ atom in the protein. Using this approach, the protein’s relative distance/positional information is also conserved.

(Equation 2) $$\begin{array}{l} \hat{X_{i}}=\frac{X_{i}-\mu_{X}}{\sigma_{X}}\\ \hat{Y_{i}}=\frac{Y_{i}-\mu_{Y}}{\sigma_{Y}}\\ \hat{Z_{i}}=\frac{Z_{i}-\mu_{Z}}{\sigma_{Z}} \end{array}$$

The overall architecture of the model can be divided into three segments: an encoder, LSTM, and a decoder. First, the encoder, which is a simple feedforward neural network, transforms all the input vector $I_{i}$ to vector $E_{i}$. Next, the LSTM segment transforms the tensor **E** into tensor **L**. It is important to note here that the LSTM output $L_{i}$ not only depends on $E_{i}$ but possibly on all $E_{j}$, where *j* = 0.1, …, *N*, and *N* is the number of amino acids in a protein. Finally, in the decoder segment, the output vectors $L_{i}$ from LSTM are transformed to $B_{i}^{p}$. $B_{i}^{p}$ is the predicted B factor of $C_{\alpha }$ at position *i* and its dimension is 1 × 1 and the dimension of tensor $B_{p}$ is *N* × 1. Functional mapping across all the segments is shown in Equation 3 as:

(Equation 3) $$\begin{array}{l} E_{i}=f_{encoder}\left(I_{i}\right)\\ L=f_{lstm}\left(E\right)\\ B_{i}^{p}=f_{decoder}\left(L_{i}\right)\\ where,i=1,2,\ldots N\;and \end{array}$$

To optimize all the parameters, the mean square error loss function in Pytorch 1.12 is used as the objective/loss function. The loss function is shown in Equation 4 and $B_{i}^{p}$, $B_{i}^{e}$ are defined as the predicted and actual B factor of $C_{\alpha }$ at $i^{th}$ position. In addition, to check the robustness of the proposed model architecture, the model is trained and tested using four different seeds, and variation in the quality of fit is observed.

(Equation 4) $$MSE=\frac{\sum_{i=1}^{N}{\left(B_{i}^{p}-B_{i}^{e}\right)}^{2}}{N}$$

### Un-normalized and normalized B factor

Previous models developed have focused on predicting either the un-normalized or normalized B-factor values. Un-normalized B factor here is referred to the B-factor value, which is directly obtained from experiments, and which can have high uncertainty depending on experimental limitations or particularly in more disordered and dynamic regions of a protein.^2^ It has been shown in the literature^2^ that the low X-ray resolution of 3–5 Å can lead to absurd B-factor values as high as 100–200 ${Å}^{2}$. Even with the fine resolution of 1.5 Å, the uncertainty in the B factor can be as high as 15%.^37^ Hence, to compare the B-factor values across different proteins/structures, it is important to normalize it.^24^ It is appropriate to normalize the B factor using Equation 5a, where $\hat{B_{i}}$, $B_{i}$ are the normalized and un-normalized B-factor values of an amino acid *i* in a protein with total *N* amino acids, respectively. $\mu_{B}$, $\sigma_{B}$ are the mean and standard deviation of un-normalized B-factor value within the same protein calculated using Equations 5b and 5c, respectively. As pointed out above, the B factor (un-normalized or normalized) is treated more as a relative property, so it is important to accurately capture the variation of the B factor within the protein. To verify if the correct trend of the B factor is captured, researchers have used PCC between the actual and predicted B factor over all residues in the protein sequence. Thus, PCC is used here as the metric for assessing the accuracy of our model.

(Equation 5a) $$\hat{B_{i}}=\frac{B_{i}-\mu_{B}}{\sigma_{B}}$$

(Equation 5b) $$\mu_{B}=\frac{\sum_{i=1}^{N}B_{i}}{N}$$

(Equation 5c) $$\sigma_{B}=\sqrt{\frac{\sum_{i=1}^{N}{\left(B_{i}-\mu_{B}\right)}^{2}}{N}}$$

In previous studies, the prediction of both kinds of B-factor data (un-normalized and normalized) has been reported. For completeness, we have trained and tested our model for both scenarios. Since the normalized B factor has been proven to be more robust against experimental noise,^2^ all results presented in the main text use normalized B-factor data. Results related to the un-normalized B factor are presented in supplemental experimental procedures in prediction of un-normalized B factor.

### Algorithm to study the impact of neighboring atoms

The B factor of an atom is known to be impacted by its neighboring atoms (in the PS and in the 3D space). To capture this effect, SOTA models^25^^,^^26^ have considered the information of neighboring amino acids in the PS within a certain window. The size of the window ($W_{s}$) is defined as the $d_{seq}$ between the first and the last amino acid in the window. To study the impact of $W_{s}$ on the overall prediction of the B factor, we perform sensitivity analysis using five different proteins. This analysis is to determine if there is one common $W_{s}$ that can be used for all the proteins.

The study with $W_{s}$ only reveals the impact of $d_{seq}$ but does not shed light on the impact of $d_{euc}$ on the B factor. To study the impact of $d_{euc}$ on predictions, $\hat{B_{i}}$ values are calculated considering only the data from $C_{\alpha }$ atoms, which are within a certain $d_{euc}$ value. The variation of PCC with $d_{euc}$ is studied to identify an appropriate $d_{euc}$ value for the proper prediction of the B factor. It should be noted that just studying the variation of PCC with $d_{euc}$ may be misleading because the number of atoms within the cutoff $d_{euc}$ is proportional to the cube of the cutoff $d_{euc}$ value. Hence, to find the impact of each atom within a cutoff $d_{euc}$, we study the variation of the ratio of the change in the PCC value (ΔPCC) for each increment of the cutoff value and total increase in the number of atoms (Δ Atoms) with respect to the cutoff $d_{euc}$. To find the cutoff $d_{euc}$, we perform the above study with 115 randomly chosen test proteins.

### Resource availability

#### Lead contact

Further information should be directed to and will be fulfilled by the lead contact, Sinan Keten (s-keten@northwestern.edu).

#### Materials availability

This study did not generate new unique materials.

## Data Availability

The authors did not generate any new data for this study but rather used the data from PDB. The codes and files necessary for training as well as testing the model are available on Open Science Framework.^38^

## Supplemental information

Document S1. Supplemental experimental procedures, Figures S1–S3, and Table S1

Document S2. Article plus supplemental information
