# Supplementary material for: B-factor prediction in proteins using a sequence-based deep learning model
Source: Patterns (N Y). 2023 Aug 4;4(9):100805. doi: 10.1016/j.patter.2023.100805 (PMC10499862; doi:10.1016/j.patter.2023.100805)
Supplement: Document S1. Supplemental experimental procedures, Figures S1–S3, and Table S1 [file mmc1.pdf]

Patterns, Volume *4*

Supplemental information

B-factor prediction in proteins using a sequence-based deep learning model

Akash Pandey, Elaine Liu, Jacob Graham, Wei Chen, and Sinan Keten

Supplemental Experimental Procedures

1 Deep Learning Model Details

	Input Dimension		Output Dimension	
Layer type	Un-normalized B-factor	Normalized B-factor	Un-normalized B-factor	Normalized B-factor
Dense	(None,28)	(None,28)	(None,32)	(None,32)
Dense	(None,32)	(None,32)	(None,64)	(None,64)
Dense	(None,64)	(None,64)	(None,64)	(None,64)
Dense	(None,64)	(None,64)	(None,64)	(None,64)
LSTM	(None,500,64)	(None,500,64)	(None,500,1024)	(None,500,1024)
Dense	(None, 1024)	(None, 1024)	(None, 512)	(None, 1024)
Dense	(None, 512)	(None, 1024)	(None, 512)	(None, 512)
Dense	(None, 512)	(None, 512)	(None, 256)	(None, 512)
Dense	(None, 256)	(None, 512)	(None, 128)	(None, 256)
Dense	(None, 128)	(None, 256)	(None, 32)	(None, 256)
Dense	(None, 32)	(None, 256)	(None, 1)	(None, 128)
Dense	-	(None, 128)	-	(None, 32)
Dense	-	(None, 32)	-	(None, 1)

Table S1: Details of the model architecture.

2 Diversity of test dataset

One of the ways to check the robustness and applicability of a machine-learning model is to study the distribution/spread of input variables. Before plotting any distribution, we introduce three new variables called the normalized amount of α helix ($\overline{a}$), β sheet ($\overline{b}$), and coil ($\overline{c}$) secondary structure. The formula to obtain $\overline{a}$, $\overline{b}$, and $\overline{c}$ for a protein with N number of amino acids is given in Eq.S1.

$$\begin{aligned}\overline{a} &= \frac{\text{Number of amino acid forming } \alpha \text{ helix}}{N} \\ \overline{b} &= \frac{\text{Number of amino acid forming } \beta \text{ sheet}}{N} \\ \overline{c} &= \frac{\text{Number of amino acid forming coil}}{N}\end{aligned} \tag{S1}$$

The information about the secondary structure of each amino acid in the protein is obtained from PDB. The probability density of N, $\overline{a}$, $\overline{b}$, and $\overline{c}$ is shown in Fig.S1. The probability density of N

shows a good spread of data in between 100-500. The distribution of $\overline{a}$ and $\overline{b}$ exhibit two peaks, and the peak at 0 signifies that some proteins might have pure alpha-helical or pure beta sheet secondary structure. $\overline{c}$ follows a Gaussian distribution with a peak around 0.45. Overall the probability density plot shows a good diversity in the data used for testing the model. To further show the diversity of the data, we randomly select 100 proteins from the test dataset and calculate the global alignment score for every pair of proteins. A global alignment finds the best alignment possible between the primary sequence of two proteins and scores it based on the number of matches, mismatches, and gaps. To keep it simple and straightforward, we use the *globalxx* command in the Biopython package to calculate the alignment score. The *globalxx* command gives 1 point for matches but does not detect any point for mismatches and gaps. The alignment score obtained is normalized by the total number of amino acids (N) in the protein. The heat map of the normalized global alignment score is shown in Fig.S2. It can be observed from the heat map that most of the alignment is poor (dark patches). However, some patches are white indicating a good alignment score but these are countable in the 100*100 area. Off-diagonal white cells do not mean that the proteins are exactly the same, it just means that after introducing gaps in the proteins in a pair, the score is good. These gaps are the mismatch that makes the proteins in a pair different. This scenario can happen, for example, when we compare proteins from the same family. The diagonal elements as expected have a normalized score of 1. But the majority of dark patches indicate bad alignment scores; hence good diversity in the test dataset.

3 Prediction of Un-normalized B-factor

In this section, we discuss the deep learning model trained on the un-normalized B-factor. To make sure that we are not dealing with very noisy data, data cleaning steps as discussed in Sec. **Dataset** were performed, but it does not guarantee that all the noisy data will be eliminated. Even though the un-normalized B-factors lie in the interval (0,80], the error values are quite low as all the B-factors were divided by 80 as it is the highest value of B-factor considered in our study as stated in Sec. **Dataset**.

The trained model is tested on 2.4k proteins and the predicted B-factor versus actual B-factor graph is shown in Fig.S3b. Fig.S3b shows the B-factor prediction for each C_α atom in 2.4k proteins, which in turn is approximately 660k C_α atoms. The PCC is calculated for each 2.4k protein and then averaged over them. The PCC on the test dataset using the fitted model is 0.73 and it is the highest correlation coefficient ever reported in the literature for un-normalized B-factor of C_α atoms. To further test the robustness of the model architecture proposed in the Sec.**Deep Learning Model**, we ran the model with 4 different seeds and it did not result in any appreciable difference in the average test PCC as it only varied between 0.732 to 0.74.

To put our work into context, we compare our results with the state-of-the-art (SOTA) models,[1] which used multiscale weighted colored graphs (MWCGs) to predict un-normalized B-factor and tested on 364 proteins using a leave-one-out strategy. To carry out a one-on-one comparison of our model with the SOTA model, we first trained our deep learning model by keeping the same 364 proteins out of the training dataset. After the training, we used the trained model and performed fine-tuning on the same model to test 364 on proteins using a leave-one-out strategy as done for the SOTA model. We obtained an average PCC of 0.7 when compared to 0.66 from the SOTA model. One of the major differences in our models is that the SOTA model uses global features like R-value and resolution which are experimental setup dependent. Conversely, we don't use any global features in our model, which makes it easier to predict the B-factor for proteins on which experimental data does not exist. Given the rapidly growing number of proteins in PDB, it is not computationally

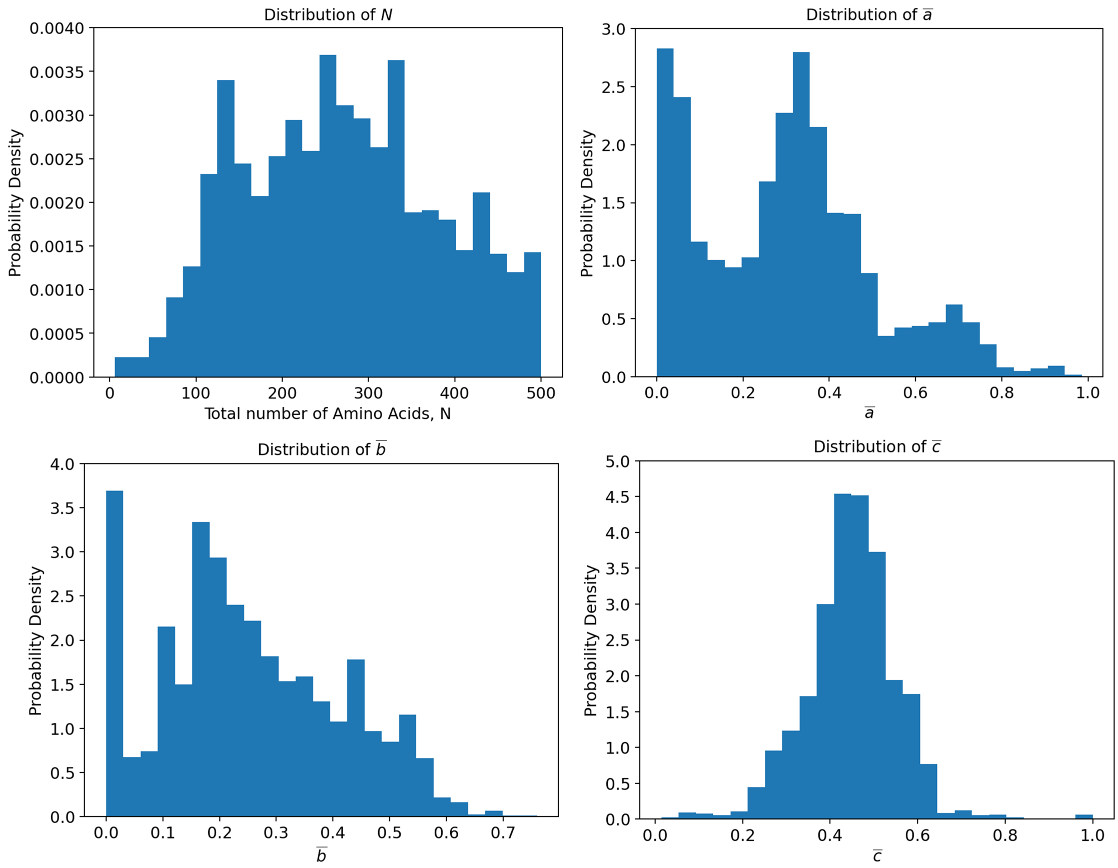

Figure S1: Probability density plot

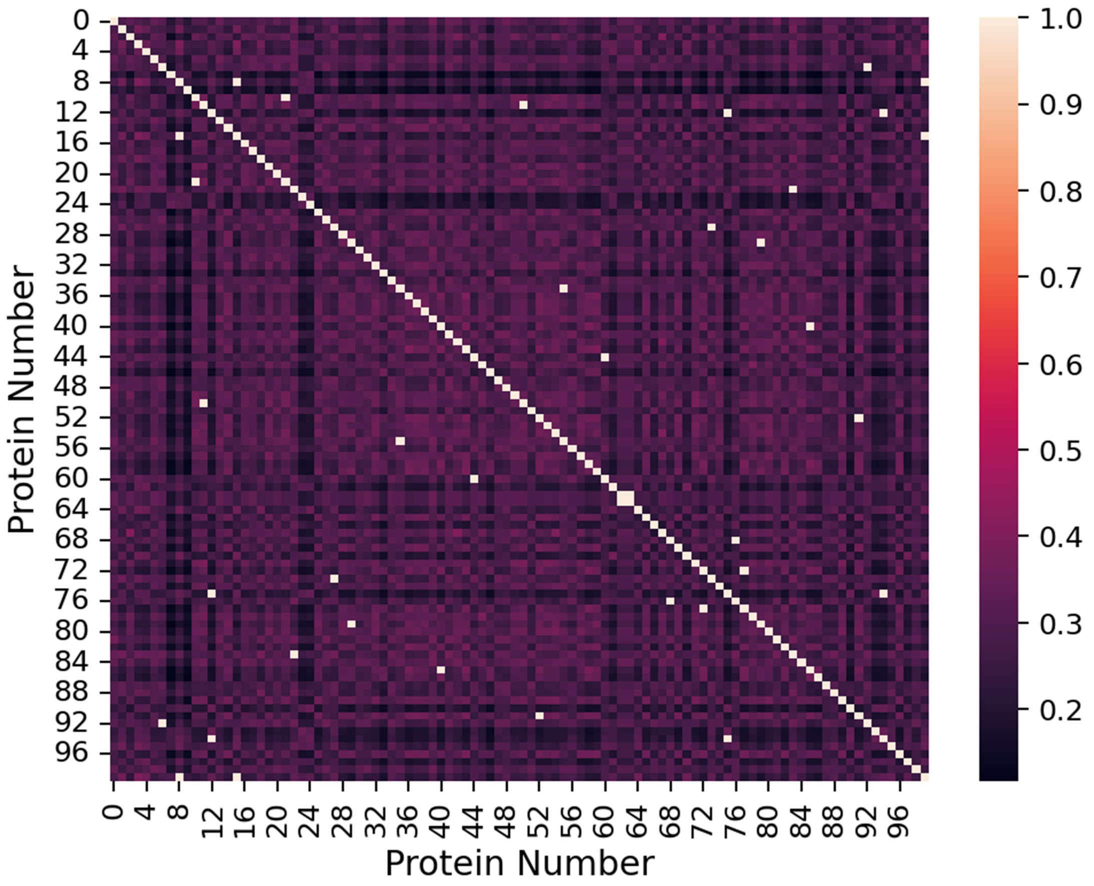

Figure S2: Normalized global alignment score

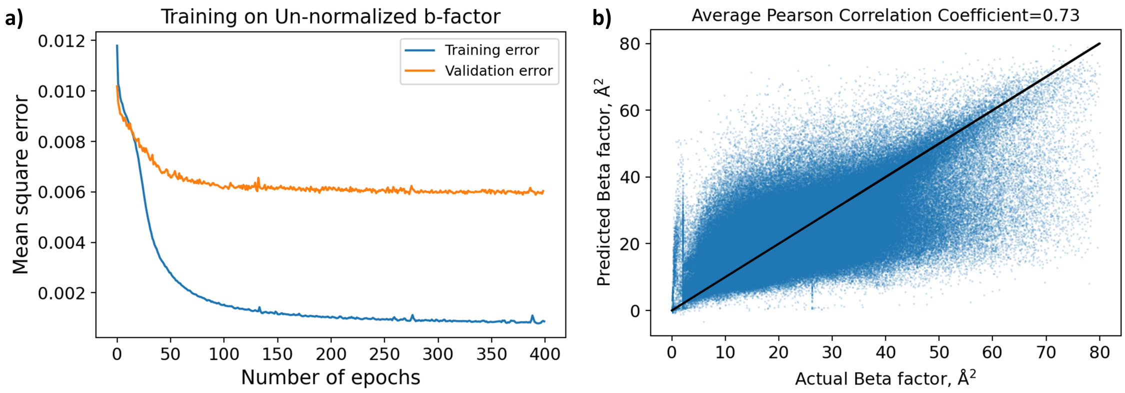

Figure S3: Model training details for un-normalized B-factor. A) Training and validation error trend for un-normalized B-factor model. B) Predicted versus actual un-normalized B-factor of atoms in 2.4k proteins in test dataset

feasible to test our model using a leave-one-out strategy. Nevertheless, testing at once on a greater number of proteins ascertains the generalizability of our model.

Supplemental References

1. Bramer, D., Wei, G.-W. (2018). The Journal of Chemical Physics, 149, 134107. 10.1063/1.5048469.
